# Dry Deposition of Ozone to Freshwater Lake Surfaces

**DOI:** 10.1021/acsestair.5c00257

**Published:** 2025-11-06

**Authors:** Audrey E. Lyp, Rebecca Z. Fenselau, Delaney B. Kilgour, Timothy H. Bertram

**Affiliations:** † Department of Chemistry, 5228 University of Wisconsin-Madison, Madison, Wisconsin 53706, United States

**Keywords:** ozone, dry deposition, freshwater lakes, dissolved organic carbon (DOC), Lake Michigan

## Abstract

The reaction of ozone (O_3_) with iodide and dissolved organic carbon (DOC) in the sea surface microlayer is a major pathway for O_3_ loss from the troposphere. The impact of O_3_ dry deposition to freshwater surfaces (e.g., inland lakes) is understudied, where current regional air quality models are unconstrained by experimental measurements of O_3_ deposition rates. Since iodide concentrations in lake water are typically negligible, O_3_ reactions at these surfaces are likely controlled by the reaction of O_3_ with DOC. This study aims to better constrain the reactive loss of O_3_ to inland waters by measuring the reactivity of O_3_ with samples collected from freshwater lakes in Wisconsin and Michigan. We find that the reactivity of O_3_ to lake water is comparable to seawater and suggest that the O_3_ dry deposition rate can be parametrized as a function of lake water DOC concentration. Calculated deposition velocities and the resulting O_3_ loss rates highlight that dry deposition to freshwater lakes reduces net production of O_3_ particularly in shallow boundary layers.

## Graphical abstract

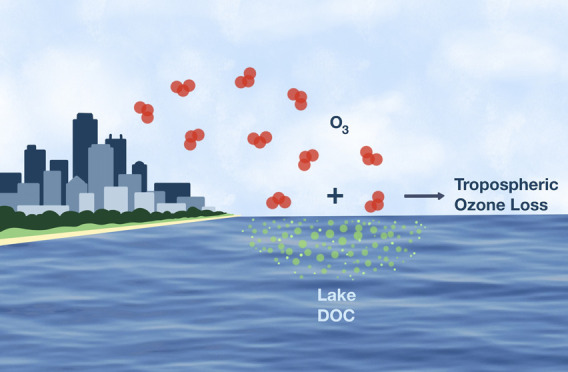

## 1 Introduction

Several large North American urban centers are located adjacent to freshwater lakes, including Chicago, Detroit, Milwaukee, and Toronto. Many of these areas are prone to experiencing poor air quality, including ozone (O_3_) exceedance events influenced by lake breeze circulation.^1^ A consequence of the lake breeze effect is that urban air containing O_3_ spends a significant amount of time in a shallow boundary layer over freshwater surfaces.^2^ As a result, there is a heightened sensitivity to the dry deposition of O_3_ in these areas. Previous model analyses of O_3_ chemistry in the Great Lakes region have conflicting viewpoints on the role of dry deposition of O_3_ over lake water. Sillman et al. find that near-total suppression of dry deposition of O_3_ over Lake Michigan is necessary to explain the high O_3_ concentrations measured.^3^ In contrast, Fast and Heilman use a model representation of the chemical production of O_3_ over Lake Michigan to find that during a 9-h daytime period, O_3_ production rates of 22 ppb h^–1^ over the lake are offset by strong deposition (−4.7 ppb h^–1^) in addition to horizontal advection and vertical transport (−7.2 ppb h^–1^).^4^

The deposition and subsequent reaction of O_3_ with iodide and dissolved organic carbon (DOC) in the surface ocean has a significant impact on the total global O_3_ budget.^5^ Despite this importance, there is considerable uncertainty in the rate and variability of O_3_ deposition to seawater as compared to other surface types (i.e, terrestrial surfaces).^5,6^ Measured O_3_ deposition velocities to seawater are reported in the range of 0.01 to 0.15 cm s^–1^.^7–11^ The range of deposition velocities observed is influenced by both physical and chemical ocean properties. O_3_ ocean deposition has been observed to increase with increasing wind speed, while deposition decreases with increasing distance from the coast, likely due to changing composition in the sea surface layer. Both modeling studies and field measurements suggest there is a low dependency of O_3_ deposition on water temperature.^7,8^ In addition to acting as an O_3_ sink, the reaction of O_3_ with DOC can also produce volatile organic compounds that further influence atmospheric processes.^12–14^

Very few measurements of O_3_ deposition to lake surfaces have been reported. Documented deposition velocities to freshwater in the literature range from 0.01 to 0.1 cm s^–1^.^7,8^ In an early study (1969), Aldaz measured a deposition velocity of approximately 0.07 cm s^–1^ to a New Mexico lake sample using the box decay method.^15^ Galbally and Roy also used the box decay method to measure a range of deposition velocities from 0.01 to 0.1 cm s^–1^ to freshwater.^16^ Wesely et al. directly measured an O_3_ deposition velocity to a lake surface using eddy covariance (EC) with reported values of approximately 0.01 cm s^–1^.^17^ Fung et al. measured deposition also using EC over a boreal lake in southern Finland with a reported value of 0.086 cm s^–1^, and found elevated deposition velocities with enhanced waterside convective mixing at night.^18^ McKay et al. spiked artificial seawater samples with humic acid (1 to 6 mg L^–1^) to measure deposition velocities in the range of 0.001 to 0.036 cm s^–1^ and observed that deposition velocity increased with increasing humic acid concentration.^19^ Additionally, Wang et al. estimate a deposition velocity of 0.0045 cm s^–1^ to riverine freshwater collected from the Pearl River.^20^

A global survey of 7514 lakes found that 87% of lakes had a near-surface DOC concentration between 80 and 1667 μM with a median of 476 μM, which is substantially higher in comparison to the range of 40–80 μM DOC observed in the surface ocean.^21,22^ Lake DOC is a highly diverse heterogeneous mix of organic compounds, including humic and fulvic acids. Sources of DOC include both in-lake processes (production by phytoplankton and macrophytes) and the lake watershed, and composition is influenced by climate, local vegetation, and season.^23^ Iodide concentrations observed in lake water are typically between 0 and 10 nM, with most lakes below 4 nM.^24,25^ This is much lower than typically observed in the surface ocean (∼100 nM), thus the deposition of O_3_ to freshwater is likely to be controlled completely by reaction with DOC.^26^ Reaction rates between O_3_ and DOC vary by several orders of magnitude. In a measurement of authentic marine DOC, Shaw and Carpenter determine a second-order rate constant for O_3_ and DOC to be 2.6 × 10^7^ M^–1^ s^–1^.^27^ Clifford et al. estimate a second-order rate constant for the reaction between O_3_ and chlorophyll of ∼6 × 10^7^ M^–1^ s^–1^ at 293 K.^28^ Reaction rates have also been determined using proxy species for DOC, with a second-order rate constant determined as 1.8 × 10^5^ M^–1^ s^–1^ for C_2_H_4_ and as 8.6 × 10^8^ M^–1^ s^–1^ for dimethyl sulfide (DMS).^29,30^

Existing chemical transport models such as the Community Multiscale Air Quality (CMAQ) model account for the enhanced deposition of O_3_ due to reactivity with iodide in seawater, with values generally ranging between 0.01 and 0.04 cm s^–1^.^31^ However, most air quality models do not include a reactive term for enhanced deposition due to O_3_ reactivity with DOC in seawater and freshwater, thus deposition is limited to the low solubility of O_3_ in water.^31^ In the absence of enhanced chemical reactivity, the O_3_ deposition velocity is typically less than 0.005 cm s^–1^ for wind speeds encountered over large freshwater lakes.^31,32^ Therefore, O_3_ deposition to freshwater is likely underestimated by current regional air quality models in shallow boundary layers.

This study presents measurements of the reactivity of O_3_ to four freshwater lakes: Lake Mendota, Lake Monona, Lake Wingra (Madison, WI), and Lake Michigan (South Haven, MI). These measurements are used to calculate a second-order rate constant for the reaction of O_3_ with DOC. We then compute the O_3_ dry deposition velocity (*v*_dep_) from the NOAA Coupled Ocean-Atmosphere Response Experiment (COARE) model and assess the sensitivity of O_3_ to *v*_dep_ (O_3_) in the Great Lakes region.

## 2 Methods

### 2.1 Sample Collection and DOC Measurements

Lake water used in laboratory ozonolysis experiments was collected nearshore from Lake Mendota, Lake Monona, Lake Wingra, and Lake Michigan during the months of October to December 2024. Sampling locations are listed in Table S1. Samples were collected by completely submerging an amber Nalgene bottle (500 or 1000 mL) in the top few inches of the lake. Bottles were cleaned before sample collection by soaking overnight in a 5% HCl bath, followed by a rinse with Milli-Q water, and three rinses with lake water immediately before sample collection. Samples were analyzed as close as possible to the initial collection date, though they were also stored unfiltered and in the dark at ∼27 °F for up to three months before ozonolysis. Three samples of Lake Mendota collected on different dates were analyzed. As lake composition is influenced by seasonal variation, this accounts for the range of DOC observed during the time frame of collection.^33^ Samples of Lake Michigan, Lake Monona, and Lake Wingra were collected on a single date. For all lakes, samples were tested between October 2024 and February 2025 to get a total of three measurements for each individual lake. A 600 nM potassium iodide (KI) solution was also prepared for analysis by dissolving KI (>99.0%, Sigma-Aldrich) in High-Performance Liquid Chromatography (HPLC) water.

To measure the DOC content, 17 mL of each lake sample was filtered using a 45 μm nylon syringe filter on the date of the ozonolysis measurement and stored by refrigeration until the date of the DOC measurement. DOC concentrations were measured using a total organic carbon analyzer (Sievers M5310 C). Though DOC degraded slightly over time with sample aging, the concentrations remained similar throughout the period of sample storage. For example, the Lake Monona sample had a measured DOC decrease of 6% between measurements on 12/19/24 and 02/05/25. This change corresponded to a measured decrease of 9% in O_3_ uptake between the two dates, demonstrating that O_3_ reactivity tracks with DOC concentration. Dates of sample collection, sample ozonolysis measurement, sample DOC concentration, and date of DOC measurement are summarized in Table 1. Though Lake Michigan samples were collected from the same sampling location, a substantial difference in DOC measurement was observed, potentially due to a strong spatial heterogeneity in near-shore sampling.

**Table 1:** Summary of Ozonolysis and DOC Experiments

Lake Sample	Date of collection	Date of ozonolysis measurement	DOC (μM C)	Date of DOC measurement
Mendota	10/24/2024	11/01/2024	469.8	11/01/2024
Mendota	11/05/2024	02/03/2025	405.6	02/05/2025
Mendota	12/10/2024	12/12/2024	391.5	12/19/2024
Michigan	11/28/2024	12/05/2024	278.3	12/19/2024
		12/05/2024	644.6	12/19/2024
		02/04/2025	448.1	02/05/2025
Monona	12/10/2024	12/12/2024	455.6	12/19/2025
		02/04/2025	426.5	02/05/2025
		02/11/2025	429.8	02/12/1025
Wingra	10/31/2024	10/31/2024	863.6	11/01/2024
		12/13/2024	641.3	12/19/2024
		02/04/2025	623.8	02/05/2025

### 2.2 Flow Tube Experimental Design

All laboratory experiments were conducted in a system modeled after the one used in Schneider et al., though modified to consist of two glass flow tubes.^34^ Both flow tubes were of the same dimensions, measuring 4.4 cm in inner diameter and 50.2 cm in length. Each flow tube contained a half-cylinder glass boat to house the sample solution. Dimensions of the glass boat were 3.5 cm in diameter and 47.8 cm in length. The maximum depth of each boat was 18 mm. The flow tube ends were sealed with a stainless-steel cap made with a Swagelok fitting used to connect to the gas flow.

O_3_ was generated by passing 16 sccm of O_2_ gas and 2000 sccm of N_2_ gas over a UV lamp (254 nm Pen-Ray Lamp; Jelight, Inc.), resulting in a typical initial O_3_ concentration of ∼85 ppb, though this concentration was subject to vary slightly due to uncontrollable lamp drift. O_3_ was then allowed to pass through the flow tubes and boats for at least 60 min prior to sample insertion to remove any potential contaminants in the system. The gas flow was evenly split between the two flow tubes, resulting in an overall flow of ∼1008 sccm through both tubes.

Two flow tubes were used in the system, one designated as a control tube to house a reference solution and the second designated to house experimental samples. The flow tube designated as the control tube was filled with 150 mL of either ultrapure Milli-Q water or HPLC-grade water (Thermo Fisher), as minimal O_3_ loss occurs to these solutions. The flow tube designated as the sample tube was filled with 150 mL of a lake water sample poured directly from the sample bottle without mixing or filtering. Measurements of O_3_ were taken continuously in 2 s intervals with UV–vis spectroscopy (Personal Ozone Monitor, 2B Technologies) in the sample tube while flow in the control tube was diverted to exhaust using Swagelok quarter-turn valves. Similarly, measurements of the O_3_ concentration in the control flow tube were made while the sample tube flow was diverted to exhaust. The schematic of this system is shown in Figure 1. System conditions are summarized in Table 2. Between experiments, sample boats were cleaned by being soaked overnight in a 5% HCl bath and then rinsed with Milli-Q water in order to remove any organic contaminants. Lab measurements are not fully representative of in situ conditions, as solutions placed into the flow tube are static as opposed to mixed and not subject to UV or thermal gradients.

**Figure 1:**
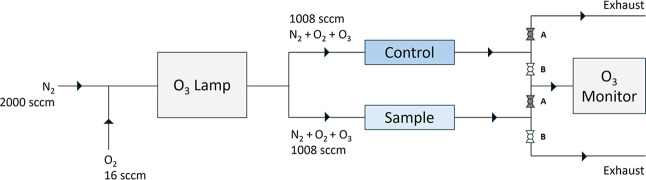
Schematic of the experimental setup. The diagram shows the flow configuration when quarter-turn valves labeled as *A* were set in the open position, allowing flow to pass through, and quarter-turn valves labeled as *B* were set in the closed position to cut off flow. This allows for O_3_ measurement of the flow to be collected through the sample tube. To measure O_3_ in the control tube flow, quarter-turn valves *A* were closed, while quarter-turn valves *B* were opened.

**Table 2:** Experimental Conditions and Constants

	Name	Value	Units
*S*	Solution surface area	166	cm^2^
*V*	Headspace volume	400	cm^3^
*t*	Residence time	23.8	s
*T*	Temperature	296	K
ω	Mean thermal velocity	3.6 × 10^4^	cm s^–1^
*H*	Henry’s law coefficient for O_3_ ^35^	8.6 × 10^–3^	M atm^–1^
*D*_O3_	Diffusion constant for O_3_ in water^36^	1.8 × 10^–5^	cm^2^ s^–1^

### 2.3 Experimental Determination of O_3_ Loss to Samples

For each lake water experiment, O_3_ loss through the sample flow tube was measured for the first 30 min of experimentation. Measurements of O_3_ were then altered between the control and sample tubes in 15 min increments for the duration of the experiment for a total experimental length of ∼105 min. Measurements of O_3_ through the control flow tube were used to determine the initial O_3_ concentration in the system before loss to the sample by calculating an interpolated average. Raw data for a typical experiment are presented in Figure 2a.

**Figure 2:**
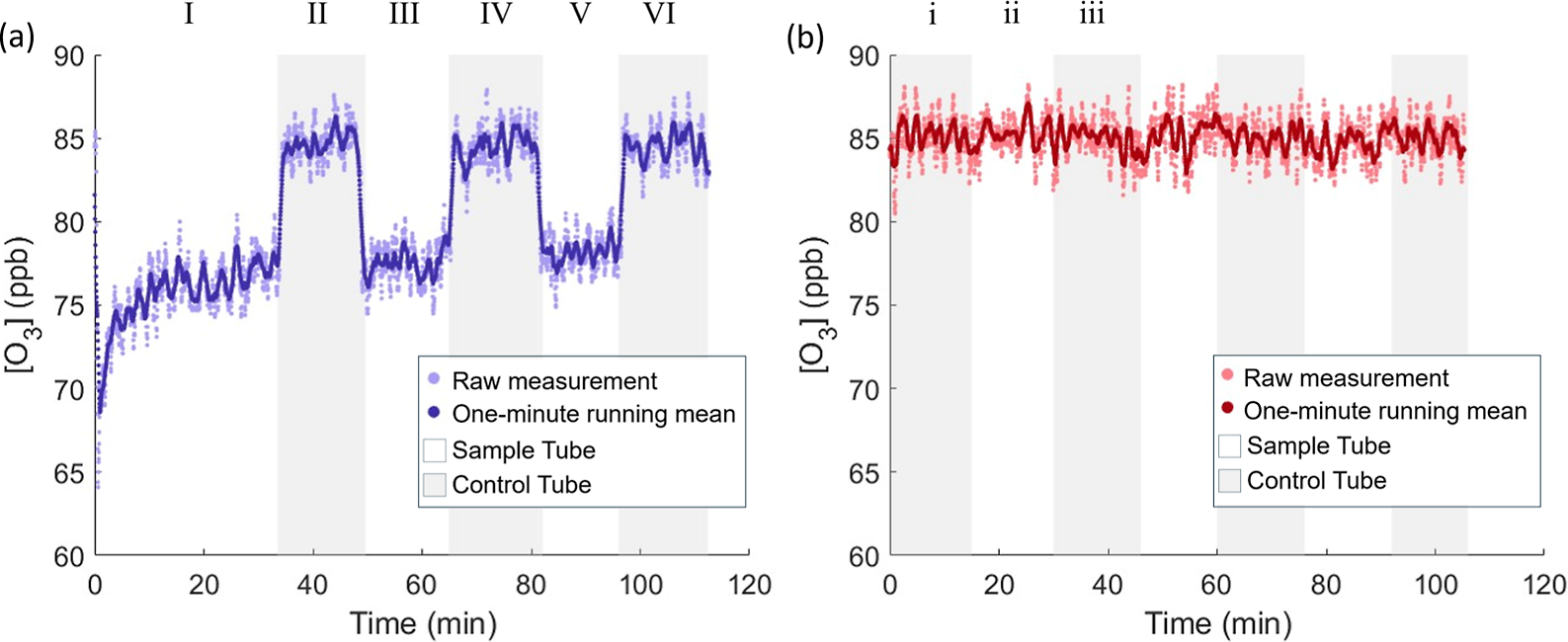
Time series of O_3_ measurements from the experimental setup. The shaded light gray areas on the plot represent O_3_ measurements taken through the control flow tube, and the white background represents O_3_ measured through the sample tube. (a) Raw data for the reaction of a Lake Mendota water sample with O_3_, representing a typical data set for lake sample experiments. (b) Raw data for experiments conducted with HPLC water in both the sample and control flow tubes.

To account for any systematic differences in the measurement of O_3_ between the sample and control flow tubes, a control solution of HPLC water was placed in both flow tubes. Measurements of O_3_ were then altered between the control and sample tubes in 15 min increments for the duration of the experiment. Raw data for a typical control experiment are presented in Figure 2b. A detailed explanation of calculated O_3_ loss is presented in the Supporting Information.

### 2.4 Calculation of Lake Uptake Coefficients

To determine an experimental first-order rate constant (*k*_exp_), initial O_3_ concentrations and O_3_ concentrations measured after loss to the sample (e.g., the pairs of interpolated O_3_ values and corrected O_3_ values as shown in Table 3) are plotted as a first-order decay using eq 1, where *t* is the flow tube residence time. Calculations are done assuming a room temperature of 296 K for all experiments.

1 $$\ln\left(\frac{{\left[O_{3}\right]}_{t}}{{\left[O_{3}\right]}_{0}}\right)=-k_{\exp}t$$

**Table 3:** Measured O_3_ Averages for Lake Mendota and HPLC Experiments

Experiment	Sample period	O_3_ (ppb)	Corrected O_3_ (ppb)	Control period	O_3_ (ppb)	Interpolated O_3_ (ppb)	ΔO_3_ (ppb)	*k*_exp_ (s^–1^)	γ
Mendota	I	76.5	76.8	II	84.8	84.8	–8.0	4.2 × 10^–3^	1.1 × 10^–6^
(02/03/25)	III	77.5	77.8	IV	84.5	84.6	–6.8	3.5 × 10^–3^	0.9 × 10^–6^
	V	78.1	78.4	VI	84.6	84.6	–6.2	3.2 × 10^–3^	0.9 × 10^–6^
HPLC	ii	85.3	n/a	i	85.3	85.2	0.1	n/a	n/a
				iii	85.1				

The experimental uptake coefficient (γ_exp_) is then calculated from the experimental first-order rate constant using eq 2 following the calculations used by Schnieder et al.^34^ Calculated uptake is dependent on the flow tube geometry used in the experiment, where *S* is the surface area of the solution inside the glass boats, *V* is the inner volume of the flow tube, and ω is the mean thermal velocity at 296 K.

2 $$\gamma_{\exp}=\frac{4k_{\exp}}{\omega \frac{S}{V}}$$

Extraction of the uptake coefficient is possible because gas-phase diffusion is not limiting for the low uptake values measured in these experiments.

## 3 Results and Discussion

### 3.1 Comparison of Observed DOC and Iodide Depletion

Prior laboratory studies have shown that the reaction of O_3_ with surface iodide results in iodide depletion in the solution for unmixed reactors.^34^ This observation is consistent with results observed in our experiment where O_3_ loss over a 600 nM KI solution was monitored over a 5 h period, as shown in Figure 3a. DOC depletion over a 5 h period was also monitored for a 429.8 μM Lake Monona sample, as shown in Figure 3b.

**Figure 3:**
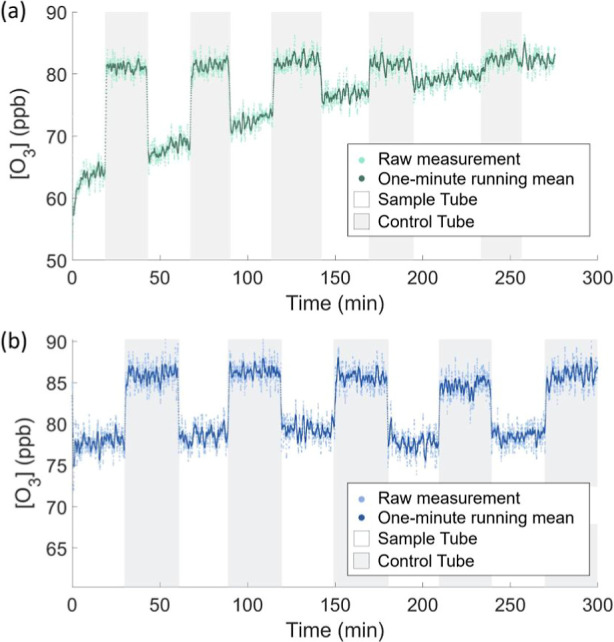
O_3_ measurements over a five-hour period for (a) 600 nM KI solution and (b) 429.8 μM Lake Monona sample. The shaded light gray areas on the plot represent O_3_ measurements taken through the control flow tube, and the white background represents O_3_ measured through the sample tube.

During the course of the KI experiment, it was observed that while O_3_ loss to the solution was substantial at the beginning of the experiment (−17.2 ppb), at the end of the experiment, O_3_ concentrations in the sample tube were nearly equivalent to those measured in the control tube (−0.3 ppb), suggesting that nearly all of the iodide in the solution surface had been depleted. In contrast, no significant reduction in O_3_ loss to Lake Monona DOC was observed, as loss values measured over the duration of the experiment were −8.1 ppb, −7.6 ppb, −6.8 ppb, −7.4 ppb, and −6.8 ppb, respectively. Additionally, a similar lack of DOC depletion to a 644.6 μM Lake Michigan sample was observed over a 3 h period. This observation suggests that the reaction rate constant for O_3_ with DOC is significantly slower than the reaction with iodide. This is consistent with reported literature second-order rate constants for iodide (2.4 × 10^9^ M^–1^ s^–1^ at 293 K) and DOC (1.8 × 10^5^ to 8.6 × 10^8^ M^–1^ s^–1^).^29,30,37^ However, as DOC is present in the surface at concentrations several orders of magnitude higher than iodide, this leads to the experimental observation of O_3_ loss on similar scales.

### 3.2 Experimental Uptake Coefficients to Lake Water

For each lake sample described in Table 1, an experimental uptake coefficient was determined. Calculations of the experimental first-order rate constant and uptake coefficients for the experiment with Lake Mendota water (Figure 2) are summarized in Table 3. The three uptake coefficients are then averaged to determine the overall uptake coefficient for the lake sample. Uptake coefficients calculated for all measured lake samples are plotted against DOC concentration in Figure 4.

**Figure 4:**
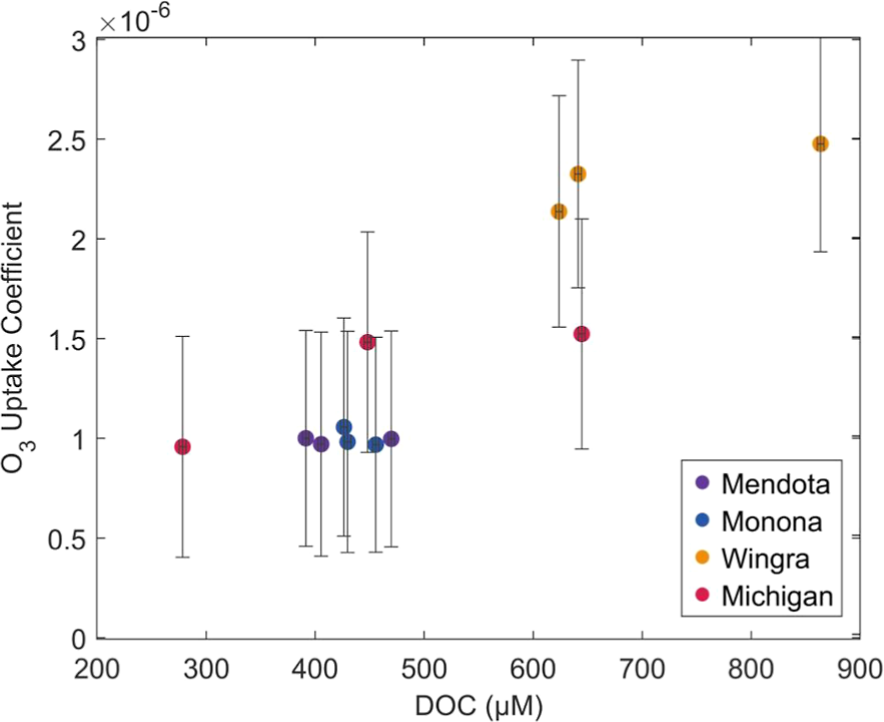
Measured O_3_ uptake coefficients for Lake Mendota, Lake Monona, Lake Wingra, and Lake Michigan samples as a function of dissolved organic carbon (DOC).

Ozone uptake coefficients are measured for lake samples spanning a range of 278.3 to 863.6 μM DOC. Lake Wingra, the smallest and most concentrated of the four lakes measured, represents an upper limit of uptake coefficients when considering dry deposition of O_3_ to the Great Lakes. Additionally, uptake coefficients are in the same range that Schnieder et al. observed for iodide concentrations between 195–780 nM.^34^

Field measurements for O_3_ loss are typically expressed in terms of a deposition velocity, which is a function of turbulent mixing and reactivity at the surface. To calculate the deposition velocity based on observed experimental uptake coefficients, a second-order rate constant (*k* ^II^) is first determined. In order to obtain this value, the linear regression of the pseudo first-order rate constant (*k* ^I^) versus DOC is calculated (Figure 5). The pseudo first-order rate constant is a separate value from the experimental first-order rate constant, as it accounts for the solubility and diffusion of O_3_ at the surface solution. To extract this relationship, the pseudo first-order rate constant is calculated from the measured uptake coefficients using eq 3, where *R* is the universal gas constant (atm M^–1^ K^–1^), *T* is temperature (296 K), *H* is the Henry’s law coefficient for O_3_, and *D*_O3_ is the liquid phase diffusion constant for O_3_, as listed in Table 2.

3 $$\gamma_{\exp}=\frac{4RTH\sqrt{D_{O_{3}}k^{I}}}{\omega }$$

**Figure 5:**
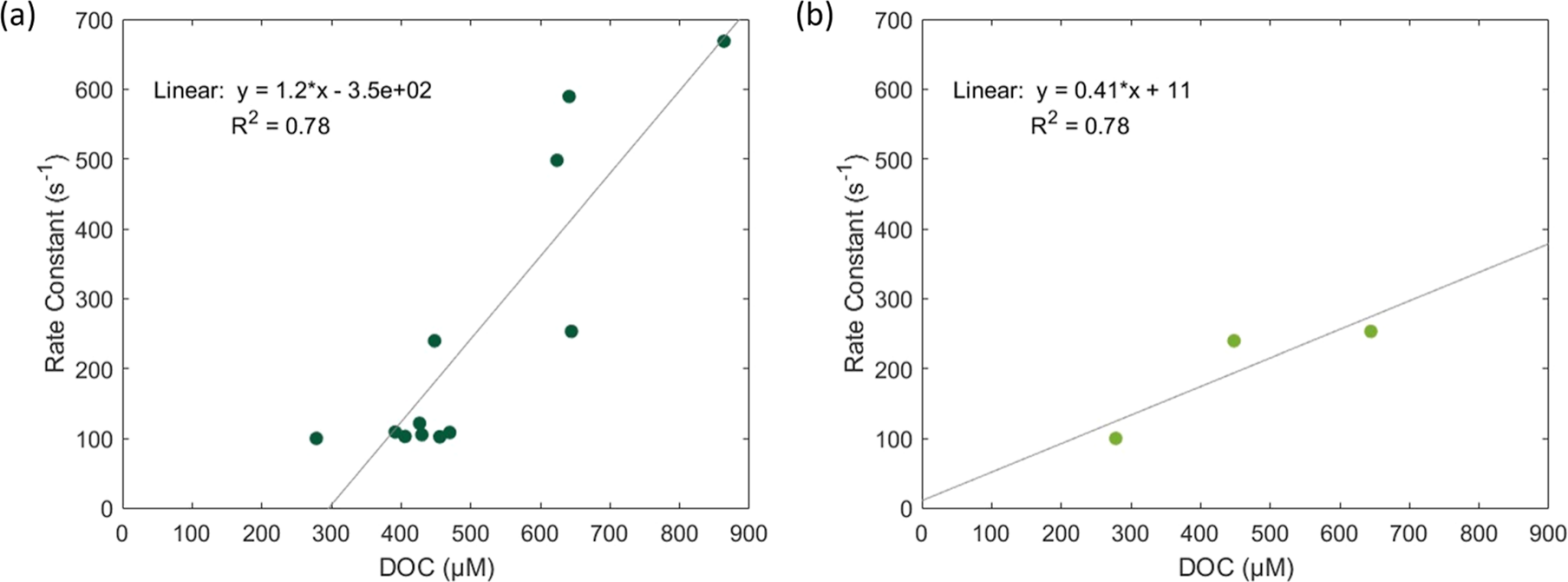
(a) Linear regression of the measured pseudo first-order rate constant for O_3_ reacting with all measured lake samples as a function of the sample dissolved organic carbon (DOC) concentration. (b) Linear regression assessed for Lake Michigan samples only.

In this calculation, the experimental uptake coefficient is assumed to be equivalent to the bulk solution uptake coefficient. While there could potentially be a DOC concentration gradient at the solution surface, we observe that the uptake coefficient does not change in time for these samples (Figure 3b), which is consistent with this approximation.

The calculated second-order rate constant for lake DOC in these experiments is 1.2 × 10^6^ M^–1^ s^–1^ (Figure 5a). While this is an order of magnitude lower than the rate constant determined for marine DOC (2.6 × 10^7^ M^–1^ s^–1^), it falls within the range of 1.8 × 10^5^ to 8.6 × 10^8^ M^–1^ s^–1^ measured previously for DOC.^29,30^ It should be noted that this rate constant should only be used within the range of DOC measured for this experiment (278.3 to 863.6 μM) as the linear fit predicts negative uptake coefficients for DOC concentrations lower than 300 μM, which is unrealistic. Therefore, a linear fit assuming that no uptake is observed when DOC concentrations are zero was also assessed by forcing the y-intercept to be zero. This analysis yields a second-order rate constant of 5.5 × 10^5^ M^–1^ s^–1^. However, it is unknown if uptake coefficients at these lower concentrations are also approximately linear or if these values potentially plateau as a large cluster of rate constants is observed at ∼ 100 cm^–1^. It is also unclear if the calculated rate constant using the forced intercept is representative of the reaction at low concentrations of DOC.

The linear regression in Figure 5a relies on the assumption that lake DOC reactivities between each of the four measured lakes are similar. However, it has not been characterized how differences in lake DOC compositions might impact the observed rate of reaction with O_3_. Lake Michigan is geographically isolated from and larger than the other three lakes measured. Therefore, a separate linear regression of only Lake Michigan data was performed. This analysis yields a second-order rate constant of 4.1 × 10^5^ M^–1^ s^–1^ to Lake Michigan DOC (Figure 5b). While this is an order of magnitude lower than the second-order rate constant calculated for all samples, it is on the same order of magnitude as the rate constant determined for C_2_H_4_.^29^ Additionally, the y-intercept is near zero, which is physically consistent since minimal uptake is expected for 0 μM DOC. This suggests that the rate constant to Lake Michigan DOC can be used to extrapolate reactivity to DOC concentrations at the lower limit.

Future work should specifically address sample collection in the lower concentration limit, as well as investigate differences in lake composition to continue to assess the impact of these factors on the DOC rate constant.

### 3.3 Model Calculation of Deposition Velocity

The atmospheric deposition velocity of O_3_ to lake water is calculated as a function of DOC in Figure 6a using the experimentally determined second-order rate constant for lake DOC of 1.2 × 10^6^ M^–1^ s^–1^ and the NOAA COARE model adapted from Fairall et al.^38^ This calculation is then repeated for Lake Michigan DOC using the second-order rate constant of 4.1 × 10^5^ M^–1^ s^–1^ in Figure 6b. In these calculations, we use a wind speed of 5 m s^–1^, a water surface temperature of 15 °C, and a friction velocity of 0.3 m s^–1^.

**Figure 6:**
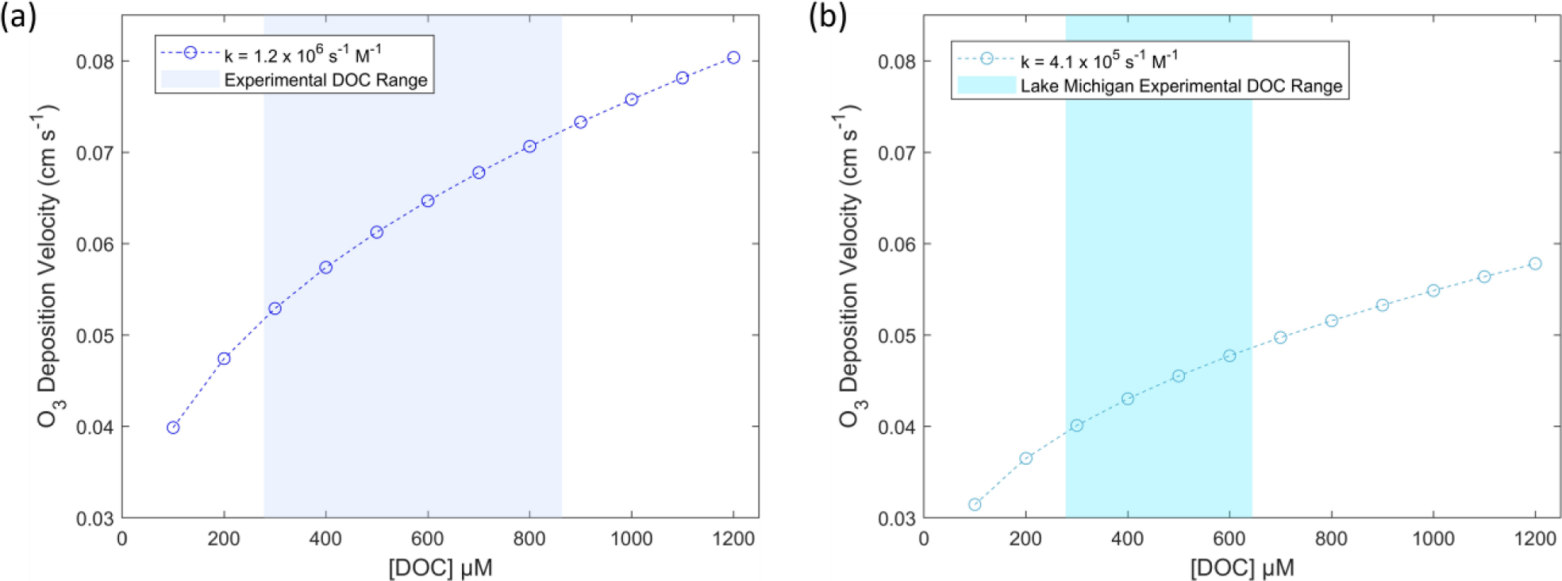
Calculated deposition velocities as determined using the NOAA COARE model for (a) all lake DOC samples and (b) only Lake Michigan DOC samples. The shaded region represents the range of DOC concentrations measured in experiments that was used to determine the second-order rate constant.

Using these parameters, the NOAA COARE model predicts deposition velocities ranging from 0.052 cm s^–1^ to 0.072 cm s^–1^ for the 280 to 860 μM DOC range, consistent with DOC concentrations measured in the experimental lake samples. In contrast, when the model is run with no chemical reactivity, the predicted deposition velocity is 0.004 cm s^–1^ for the same DOC range. The values determined here, accounting for the chemical reactivity of O_3_ with DOC, fall within the deposition velocity range of 0.01 cm s^–1^ to 0.1 cm s^–1^ measured over freshwater surfaces reported by Ganzeveld et al.^7^ The measured range is slightly below the deposition velocity of 0.086 cm s^–1^ measured over a boreal lake by Fung et al. DOC concentration of the boreal lake is taken to be 1166 μM, though this value is based on measurements taken a year after experimentation.^18^ Our model would predict a deposition velocity of 0.079 cm s^–1^ for this concentration, which, while lower than the reported deposition velocity, still falls within reasonable agreement. The measured deposition velocities of 0.052–0.072 cm s^–1^ to lake water are higher than globally averaged modeled deposition velocities reported to the marine surface, which fall between 0.02 and 0.039 cm s^–1^.^5^ However, to the best of our knowledge, deposition of O_3_ to lake and marine surfaces are not necessarily comparable cases, as the differences in DOC composition are likely to have different reactivity in addition to the presence of iodide in the sea surface.

DOC concentrations of large freshwater lakes are lower than median values for global lakes, though existing measurements are limited.^39^ Ogorek et al. report average DOC measurements for each of the five Great Lakes (Erie, Huron, Michigan, Ontario, and Superior) that range from 105 to 209 μM. The average DOC concentration in Lake Michigan was 155 ± 11 μM.^40^ Biddanda and Cotner report an average DOC of 180 μM for Lake Michigan, which is similar to this value.^41^ Biddanda and Cotner also report Lake Michigan DOC concentrations inversely related to increasing distance from a southern river mouth. In March 2000, river water DOC was 680 μM, nearshore water DOC was 290 μM, and lake water DOC was 140 μM. In May 2000, river water DOC was 410 μM, nearshore water DOC was 127 μM, and lake water DOC was 122 μM.^42^ This demonstrates that the gradient in DOC concentration from the shore to the center of the lake will likely impact the overall O_3_ deposition observed to the lake. The March 2000 nearshore water measurement of 290 μM DOC is similar to the 278.3 μM DOC measured in the Lake Michigan sample from this study, which was also collected from nearshore waters. The Lake Michigan sample in this study was collected a few yards from the shore adjacent to where the Black River mouth feeds into the lake, which could also contribute to why the measured DOC concentration is higher than the mean literature values.

This suggests that the calculated deposition velocity of 0.052 cm s^–1^ for 300 μM DOC is potentially representative of elevated DOC concentrations observed in Lake Michigan. This deposition velocity is likely an upper limit, as the deposition velocity for 300 μM DOC calculated using only the Lake Michigan samples is 0.040 cm s^–1^. Furthermore, since we propose that the rate constant used in Figure 6b is applicable to DOC concentrations lower than experimentally measured, this corresponds to a deposition velocity of 0.034 cm s^–1^ for 150 μM Lake Michigan DOC.

### 3.4 Ozone Loss over Lake Michigan

In order to assess the sensitivity of Lake Michigan to the determined deposition velocities, we calculated the O_3_ loss rate (ppb hr^–1^) over the lake using eq 4.

4 $$L\left(O_{3}\right)=\frac{v_{dep}}{h}\left[O_{3}\right]$$

The O_3_ loss rate is determined as a function of the O_3_ concentration [O_3_] over the lake, the height (*h*) of the boundary layer over the lake’s surface, and the O_3_ deposition velocity. Vermeuel et al. describe a lake breeze event over Lake Michigan on June 2, 2017, and report an initial measured O_3_ concentration of 49 ppb in the plume as it departs the Chicago area and enters over the lake surface at 9:00 CDT.^43^ This value is incorporated as the initial O_3_ concentration for this calculation. It takes approximately 8 h for the O_3_ plume to travel north from the Gary-Chicago area to Zion, IL (890 m west of the Lake Michigan shoreline), where the plume reaches a measured value of 89 ppb O_3_ at 17:00 CDT.^43^ Plume trajectory and modeled O_3_ concentrations during the lake breeze event from the Chicago area to Zion, IL, over Lake Michigan are described in Figures S2 and S3. During the 2017 Lake Michigan Ozone Study, high levels of O_3_ were observed in a boundary layer of approximately 50 to 370 m above the lake surface, with boundary layer height dependent on location, date, and time of day.^44^ To account for this range, three model heights of 50, 100, and 370 m were used to determine the theoretical dependence of O_3_ loss on the boundary layer height. Four representative velocities were also used in this calculation. The deposition velocity of 0.005 cm s^–1^ was selected to estimate a deposition velocity for O_3_ to freshwater in current air quality models. The values of 0.052 cm s^–1^ and 0.072 cm s^–1^ were also selected to represent the lower and upper limits of deposition velocity over lake water as calculated in Figure 6a. The value of 0.034 cm s^–1^ as calculated in Figure 6b for mean Lake Michigan DOC (150 μM) was also included. Figure 7a summarizes the expected O_3_ loss rate as dependent on these parameters.

**Figure 7:**
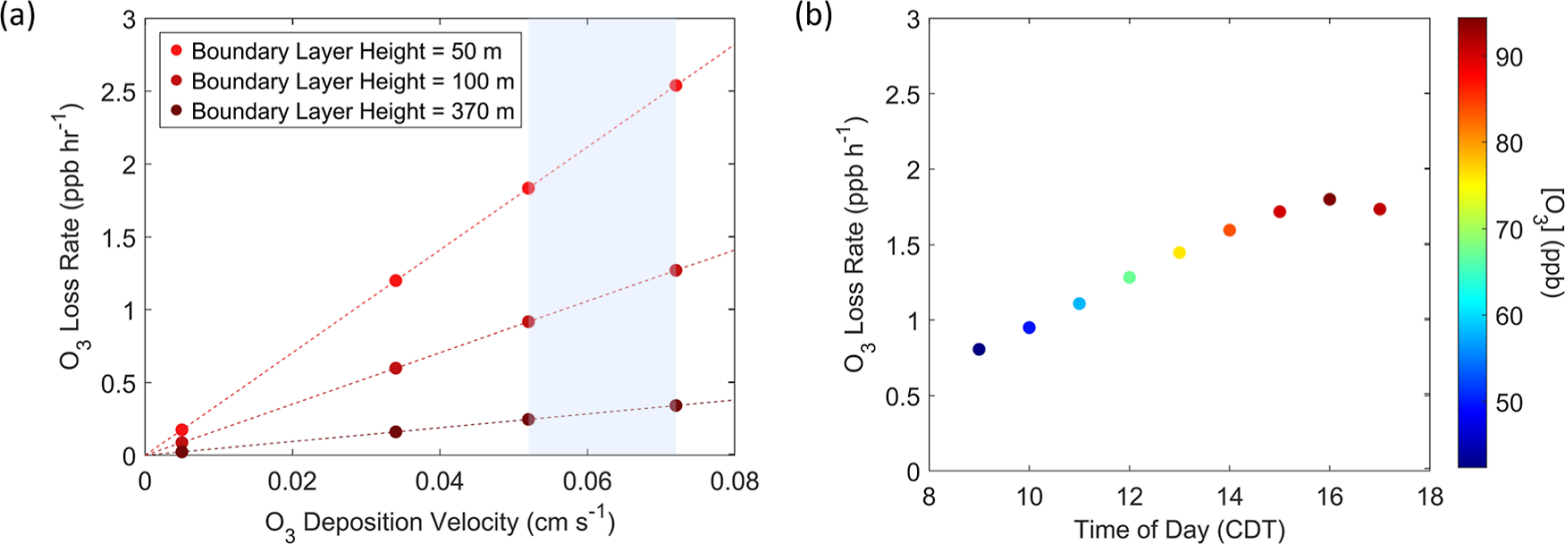
(a) Calculated O_3_ loss rates over Lake Michigan at an initial O_3_ concentration of 49 ppb. O_3_ loss is calculated for four deposition velocities (0.005, 0.034, 0.052, and 0.072 cm s^–1^) over a range of three boundary layer heights (50, 100, and 370 m). The shaded region represents the range of deposition velocities calculated from the DOC concentrations measured in these experiments. (b) Calculated theoretical O_3_ loss based on modeled O_3_ concentrations from 9:00 to 17:00 CDT for the June 2, 2017 plume over Lake Michigan as described by Vermeuel et al.^43^ The boundary layer height and deposition velocity are set at 100 m and 0.052 cm s^–1^, respectively.

Results from this analysis suggest that substantial loss of O_3_ to Lake Michigan could occur during lake breeze events within the range of expected boundary layer heights and deposition velocities over lake water determined by this study. Previous measurements of the boundary layer over Lake Michigan suggest that the diurnal profile is comparable to that of typical ocean surfaces, though with a smaller peak observed around midday.^45^ For example, in the case where the boundary layer height is increased to 370 m, an O_3_ loss of −0.2 to −0.3 ppb h^–1^ is predicted for deposition velocities within the range of 0.034 to 0.072 cm s^–1^. When the deposition velocity is set at 0.052 cm s^–1^ in a 100 m boundary layer, an O_3_ loss of −0.9 ppb h^–1^ is calculated. An upper limit of O_3_ loss of −2.5 ppb h^–1^ is determined by the shallowest boundary layer (50 m) and maximum deposition velocity (0.072 cm s^–1^).

Vermeuel et al. used a trajectory-based chemical box model over Lake Michigan to describe evolving O_3_ concentrations over the 8 h period as the plume travels from the Gary-Chicago area to Zion, IL.^43^ Hourly values for the concentrations of O_3_ reported in Vermeuel et al. were substituted into eq 4 with a set boundary layer height of 100 m and deposition velocity of 0.052 cm s^–1^ in order to determine how the loss rate of O_3_ changes in time as the plume travels over Lake Michigan, shown in Figure 7b. From this calculation, the modeled total loss of O_3_ to Lake Michigan over this 8 h period of time would be −12.2 ppb. When the calculation is applied to a deposition velocity of 0.034 cm s^–1^, this yields a total O_3_ loss of −8.0 ppb. If the same calculation were performed not accounting for enhanced O_3_ loss (i.e., using a deposition velocity of 0.005 cm s^–1^), the O_3_ loss to Lake Michigan is −1.2 ppb. This suggests that regional air quality models that do not account for the reactivity of O_3_ with DOC significantly underestimate the loss of O_3_ to freshwater surfaces. Including deposition to lake surfaces in regional models, particularly large lakes under high O_3_ conditions, would account for these enhanced O_3_ loss rates from the troposphere. Vermeuel et al. report net production rates of O_3_ between 1 to 10 ppb h^–1^ over the duration of the plume. These production rates account for O_3_ deposition to the lake surface by including an O_3_ loss term represented by a deposition velocity of 0.05 cm s^–1^.^43^

The ultimate observed loss of O_3_ to Lake Michigan will depend on the complexity of parameters such as wind speed, location, changing surface DOC concentration, boundary layer height, and atmospheric O_3_ concentration. However, these results show that it is plausible to expect that O_3_ is reactive at freshwater surfaces and that enhanced deposition should be accounted for in regional air quality models.

## Supplementary Material



## Notes

Lake sampling locations, calculation of O_3_ loss to lake samples, NOAA COARE model, and conditions for the lake breeze event described over Lake Michigan (PDF)
