# Supplementary material for: Dry Deposition of Ozone to Freshwater Lake Surfaces
Source: ACS EST Air. 2025 Nov 6;2(12):2945–53. doi: 10.1021/acsestair.5c00257 (PMC12706727; doi:10.1021/acsestair.5c00257)
Supplement: Supplementary file 1 [file ea5c00257_si_001.pdf]

Supporting Information for:

# Dry Deposition of Ozone to Freshwater Lake Surfaces

Audrey E. Lyp[1], Rebecca Z. Fenselau[1], Delaney B. Kilgour[1], and Timothy H. Bertram[1]*

[1]Department of Chemistry, University of Wisconsin-Madison, Madison WI 53706, USA

*Corresponding author: timothy.bertram@wisc.edu

## S1. Sample Locations

The latitude and longitude for the sample collection locations are shown in Table S1.

**Table S1. Coordinates for Sample Collection**

| Lake Sample | Date of Collection | Latitude | Longitude |
|---|---|---|---|
| Mendota | 10/24/2024 | 43°04’39” N | 89°24’00” W |
| Mendota | 11/05/2024 | 43°04’39” N | 89°24’00” W |
| Mendota | 12/10/2024 | 43°04’39” N | 89°24’00” W |
| Michigan | 11/28/2024 | 42°24’02” N | 86°17’07” W |
| Monona | 12/10/2024 | 43°04’09” N | 89°22’57” W |
| Wingra | 10/31/2024 | 43°03’28” N | 89°24’51” W |

## S2. Calculation of $O_3$ Loss to Samples

To determine $O_3$ loss over lake samples, the average $O_3$ concentration through the sample flow tube was determined for three timeframes over the duration of the experiment. This is represented in Figure 2a, where the average $O_3$ was calculated for periods *I, III*, and *V*. To determine this value for period *I*, data was averaged after the initial measurement stabilized, approximately fifteen minutes into the experiment. For all other periods, the entire period timeframe was used to calculate the average $O_3$ excluding the sharp changes in concentration observed as measurements were changed between flow tubes.

Similarly, the average $O_3$ concentration was determined for the control flow tube in period *II*, *IV*, and *VI* of Figure 2a. As these measurements represent the $O_3$ concentration before reaction in the system, values were interpolated by averaging the two control measurements on either end

of a sample measurement to determine the best estimation for the initial $O_3$ during the sample measurement timeframe. For example, the average of the measurements during periods *II* and *IV* was calculated to determine an initial $O_3$ concentration to compare to the $O_3$ concentration measured over the lake sample during period *III*. Likewise, the average $O_3$ of periods *IV* and *VI* was determined to compare to the $O_3$ concentration measured during period *V*.

Since measurements were collected first over the sample tube to capture the initial change in $O_3$, the same calculation could not be used for period *I*. Instead, the $O_3$ concentration determined for period *I* was compared directly to the average $O_3$ concentration determined for period *II*. Averages and interpolated values used to determine $O_3$ loss are presented in Table 3.

As $O_3$ loss was calculated as the difference between the control and sample flow tubes, control experiments were conducted to assess if a systematic offset in $O_3$ measurement between the two flow tubes was present. Identical solutions of HPLC grade water were placed in both the sample and control flow tubes as a minimally reactive water control. In Figure 2b, $O_3$ in the sample flow tube was determined by calculating the average during period *ii*. $O_3$ in the control flow tube was also averaged for periods *i* and *iii*. The average of these values was then taken to calculate an interpolated value of $O_3$ in the control tube during period *ii*. The average $O_3$ of the sample flow tube measurement (i.e., period *ii*) was then subtracted from this interpolated value for the control flow tube to determine the offset in $O_3$ measurement between the two tubes, as shown in Table 3. This calculation was repeated for the course of the experiment and several repetitions of this experiment. Collected data was then used to generate a distribution of the offset between the two flow tubes, as shown in Figure S1. The mean of this histogram was -0.3 ppb of $O_3$, indicating that on average, $O_3$ measurements through the sample flow tube were slightly lower than through the control flow tube, though the overall concentration is expected to be the same in each tube. Experiments to generate this histogram were conducted during February 2025, thus this correction was applied to all ozonolysis experiments also conducted during this timeframe. The width of the distribution was used to calculate a standard deviation for all ozone measurements, which was used to calculate the error bars in Figure 4.

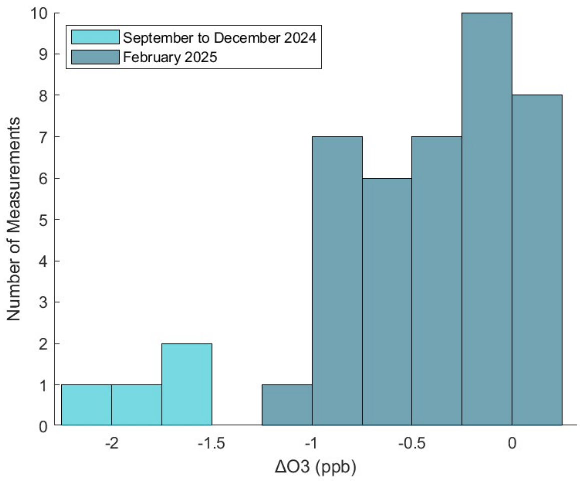


**Figure S1.** Offset calculated between sample and flow tubes.

An additional four data points were collected from September to December 2024, shown in Figure S1. In these experiments, ultrapure Milli-Q water was used in both flow tubes as opposed to HPLC grade water. The use of Milli-Q over HPLC water is expected to have minimal impact on results as offset was always calculated between two identical samples. These data points were centered around -1.8 ppb $O_3$, indicating that a shift must have occurred in the system between December 2024 and February 2025. The exact cause of this shift is unknown as no significant alterations were made to the overall system during this time.

To account for this shift and offset between the two flow tubes, +0.3 ppb $O_3$ was added to all sample measurements taken during February 2025 and +1.8 ppb $O_3$ was added to all sample measurements taken during September to December 2024. An example of how this correction was applied to the data in Figure 2a is shown in Table 3.

## S3. Description of NOAA COARE Model

The NOAA COARE model is a bulk air-sea flux algorithm developed from the international TOGA-COARE field study conducted from November 1992 to February 1993 over the Pacific Ocean. The model accounts for the air-sea transfer of atmospheric gases, including $O_3$, based on airside and waterside transfer velocities and influenced by physical conditions including wind speed, temperature, and wave effects.[1] While the NOAA COARE model is designed for air-

sea fluxes, the physics of the gas-transfer model are consistent with the physics expected of gas-transfer to lake surfaces.

## S4. Lake Breeze Event over Lake Michigan on June 2, 2017

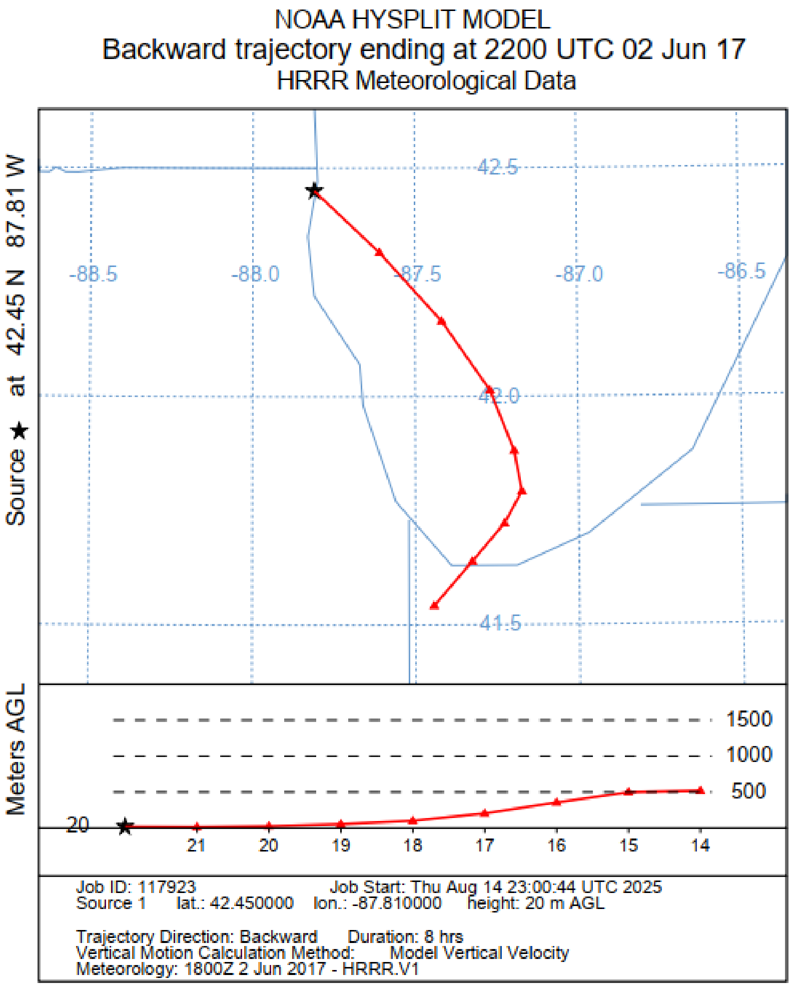


**Figure S2.** NOAA HRRR backward air mass trajectory calculations for the lake breeze event on June 2, 2017 as described by Vermeuel et al.[2] The trajectory line depicts $O_3$ plume departure from the Chicago area at 9:00 CDT (14:00 UTC) and arrival in Zion, IL at 17:00 CDT (22:00 UTC) depicted by the star.

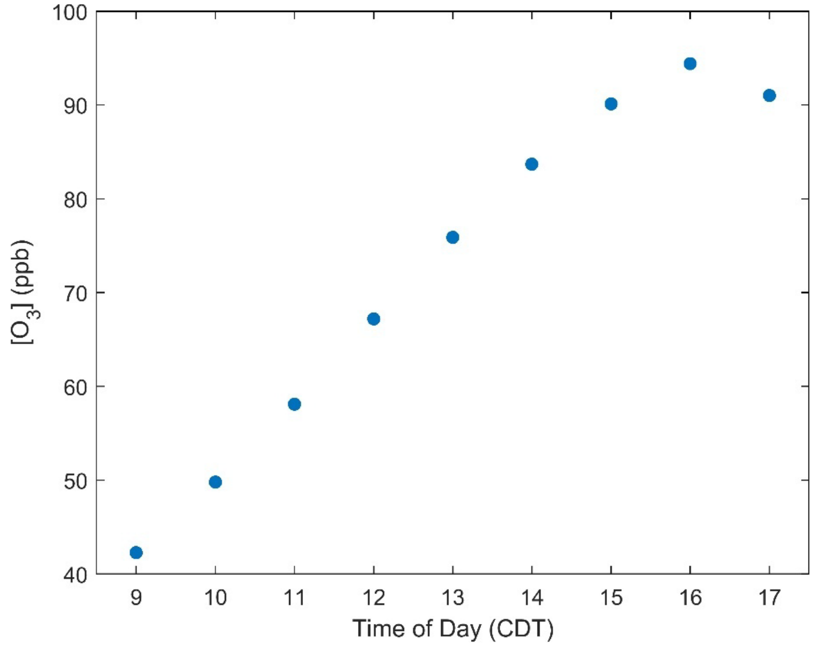


**Figure S3.** Modeled hourly $O_3$ concentrations for the lake breeze event on June 2, 2017 calculated by Vermeuel et al. as the plume departs from the Chicago area at 9:00 CDT and arrives in Zion, IL at 17:00 CDT.[2]

## References


(1) Fairall, C. W.; Yang, M.; Bariteau, L.; Edson, J. B.; Helmig, D.; McGillis, W.; Pezoa, S.; Hare, J. E.; Huebert, B.; Blomquist, B. Implementation of the Coupled Ocean-Atmosphere Response Experiment Flux Algorithm with CO2, Dimethyl Sulfide, and O3. *J. Geophys. Res. Oceans* **2011**, *116* (C4). https://doi.org/10.1029/2010JC006884.

(2) Vermeuel, M. P.; Novak, G. A.; Alwe, H. D.; Hughes, D. D.; Kaleel, R.; Dickens, A. F.; Kenski, D.; Czarnetzki, A. C.; Stone, E. A.; Stanier, C. O.; Pierce, R. B.; Millet, D. B.; Bertram, T. H. Sensitivity of Ozone Production to NO and VOC Along the Lake Michigan Coastline. *J. Geophys. Res. Atmospheres* **2019**, *124* (20), 10989–11006. https://doi.org/10.1029/2019JD030842.
